# How rare and common risk variation jointly affect liability for autism spectrum disorder

**DOI:** 10.1186/s13229-021-00466-2

**Published:** 2021-10-06

**Authors:** Lambertus Klei, Lora Lee McClain, Behrang Mahjani, Klea Panayidou, Silvia De Rubeis, Anna-Carin Säll Grahnat, Gun Karlsson, Yangyi Lu, Nadine Melhem, Xinyi Xu, Abraham Reichenberg, Sven Sandin, Christina M. Hultman, Joseph D. Buxbaum, Kathryn Roeder, Bernie Devlin

**Affiliations:** 1 Department of Psychiatry, University of Pittsburgh School of Medicine, Pittsburgh, PA USA; 2 Seaver Autism Center for Research and Treatment, Icahn School of Medicine at Mount Sinai, New York, NY USA; 3 Department of Psychiatry, Icahn School of Medicine at Mount Sinai, New York, NY USA; 4 Department of Health Sciences Department, School of Sciences, European University Cyprus, Nicosia, Cyprus; 5 The Mindich Child Health and Development Institute, Icahn School of Medicine at Mount Sinai, New York, NY USA; 6 Friedman Brain Institute, Icahn School of Medicine at Mount Sinai, New York, NY USA; 7 Department of Medical Epidemiology and Biostatistics, Karolinska Institutet, Stockholm, Sweden; 8 Department of Statistics, University of Michigan, Ann Arbor, MI USA; 9 Present Address: Genebox, Beijing, China; 10 Department of Genetics and Genomic Sciences, Icahn School of Medicine at Mount Sinai, New York, NY USA; 11 Department of Neuroscience, Icahn School of Medicine at Mount Sinai, New York, NY USA; 12 Department of Statistics, Carnegie Mellon University, Pittsburgh, PA USA; 13 Computational Biology Department, Carnegie Mellon University, Pittsburgh, PA USA

**Keywords:** Genomic-Best Linear Unbiased Prediction (G-BLUP), De novo mutation, Liability, Polygenic risk score, Autism spectrum disorder

## Abstract

**Background:**

Genetic studies have implicated rare and common variations in liability for autism spectrum disorder (ASD). Of the discovered risk variants, those rare in the population invariably have large impact on liability, while common variants have small effects. Yet, collectively, common risk variants account for the majority of population-level variability. How these rare and common risk variants jointly affect liability for individuals requires further study.

**Methods:**

To explore how common and rare variants jointly affect liability, we assessed two cohorts of ASD families characterized for rare and common genetic variations (Simons Simplex Collection and Population-Based Autism Genetics and Environment Study). We analyzed data from 3011 affected subjects, as well as two cohorts of unaffected individuals characterized for common genetic variation: 3011 subjects matched for ancestry to ASD subjects and 11,950 subjects for estimating allele frequencies. We used genetic scores, which assessed the relative burden of common genetic variation affecting risk of ASD (henceforth “burden”), and determined how this burden was distributed among three subpopulations: ASD subjects who carry a potentially damaging variant implicated in risk of ASD (“PDV carriers”); ASD subjects who do not (“non-carriers”); and unaffected subjects who are assumed to be non-carriers.

**Results:**

Burden harbored by ASD subjects is stochastically greater than that harbored by control subjects. For PDV carriers, their average burden is intermediate between non-carrier ASD and control subjects. Both carrier and non-carrier ASD subjects have greater burden, on average, than control subjects. The effects of common and rare variants likely combine additively to determine individual-level liability.

**Limitations:**

Only 305 ASD subjects were known PDV carriers. This relatively small subpopulation limits this study to characterizing general patterns of burden, as opposed to effects of specific PDVs or genes. Also, a small fraction of subjects that are categorized as non-carriers could be PDV carriers.

**Conclusions:**

Liability arising from common and rare risk variations likely combines additively to determine risk of any individual diagnosed with ASD. On average, ASD subjects carry a substantial burden of common risk variation, even if they also carry a rare PDV affecting risk.

**Supplementary Information:**

The online version contains supplementary material available at 10.1186/s13229-021-00466-2.

## Background

The genetic architecture of autism spectrum disorder (ASD) remains uncertain, although there are putative models [1]. One model posits that ASD is heterogeneous (heterogeneity model), that severe mutations in any of a large set of genes are sufficient to cause the disorder. Another emphasizes the major role played by common variation—shared by all of us to a greater or lesser extent (infinitesimal model)—in the documented high heritability of ASD [2–6]. A hybrid model asserts that common and rare variations combine in some way, perhaps additively [7], to confer liability [1, 7, 8]. At the level of a population, common variation probably plays the dominant role in liability, whereas a rare mutation can make the largest contribution to liability for an individual who carries it [9]. Still, our current understanding of the genetic architecture of ASD is unsatisfactory, especially regarding how common and rare variations jointly confer risk. This architecture is important because it has clinical consequences; for example, it could require more nuanced evaluation of recurrence risk. Establishing the exact nature of the interplay between common and rare risk variations will be challenging, however, because of the multiplicity of plausible models that could fit the current data.

The infinitesimal and additive models differ largely in the magnitude of variants’ impact on liability. Furthermore, because a strict infinitesimal model does not fit the empirical ASD data well, due to documented large effects from some rare variants, we only consider the additive model here. Notably, while heterogeneity and additive models are fundamentally different, they can share key elements, as a recent study by Oetjens and colleagues [10] describes. For subjects carrying mutations for one of 11 rare genetic disorders, Oetjens and colleagues show that quantitative traits associated with these disorders vary substantially as a function of the common genetic variation they carry and they speculate that these rare and common variants could act additively to affect the traits. Yet, they note that it would be hard to distinguish additive from non-additive models even for these quantitative traits without large data sets (see also [11, 12]). In the context of ASD and its binary diagnosis, distinguishing heterogeneity and additive models will be even more challenging.

To address this problem empirically, a sample of ASD subjects who have been characterized for rare and common variations is essential. Because rare, de novo potentially damaging mutations, henceforth PDVs, carry the most readily detectable signal for ASD association [13–16], the ideal sample would be characterized for such PDVs. Following the tradition in human genetics, we call ASD subjects carrying such PDVs as “PDV carriers” and all other ASD subjects as “non-carriers.” Such well-characterized samples of the population are not common and none as yet are especially large, a limiting factor for any study. For the sample to be analyzed here, we combine data from three sources: subjects diagnosed with ASD from the Simons Simplex Collection or SSC [17]; ASD and unaffected subjects from the Population-Based Autism Genetics and Environment Study or PAGES [9]; and subjects from the Electronic MEdical Records and Genomics Network or eMERGE [18], whom we assume have not been diagnosed with ASD and are non-carriers.

These data could be analyzed in at least two ways. One approach would be to use polygenic risk scores (PRS), which are based on common variants putatively affecting liability [19]. Typically, these variants are identified from genome-wide association studies (GWAS) and the PRS for each subject is computed as a weighted sum of the count of risk alleles they carry. Then, values of the PRS in ASD PDV carriers (ASD-PDV) and ASD non-carriers (ASD-NO-PDV), as well as unaffected subjects, can be contrasted to assess how common and rare variations jointly confer risk. An elegant version of this approach is the pTDT or polygenic Transmission Disequilibrium Test [7], which requires parental genotypes. Because only a portion of our data have parental genotypes, here we concentrate on the PRS.

The PRS is only as effective as the information arising from GWAS, which for ASD is still relatively limited compared to other phenotypes (see Grove and colleagues [6] versus the Psychiatric Genomics Consortium for schizophrenia and bipolar disorder [20, 21]). For this reason, we emphasize here another approach to developing a score, which will be based on the theory of Genomic-Best Linear Unbiased Prediction (G-BLUP) [22–24]. The ideas behind G-BLUP are similar to the PRS. Rather than using GWAS results, G-BLUP develops a predictive model to distinguish case versus control subjects, genetically, using genetic variation across the genome (see Additional File 1 for more details on G-BLUP). Here, we call this genomic prediction “GP.” If effective, the PRS and GP will not be strictly independent. Yet, if they were not *strongly dependent*, they could be combined to produce an even more effective predictor. To allow for this possibility, we tune GP to the population samples used in this study, whereas we use PRS based on different samples. Moreover, because we use a pruning and thresholding approach to the PRS, we chose a threshold that forced the number of SNPs included in the score to be relatively sparse, yet informative, and thereby limiting its correlation with GP. Note that our purpose here is not to compare the predictions of GP and PRS, the former tuned to the population sample and the latter not, but rather to develop predictors useful for examining how common and rare variations jointly confer risk of ASD.

We use these approaches to document (1) that the burden of risk variants carried by ASD subjects is stochastically greater than that carried by control subjects; (2) that carriers of rare, PDVs bear a burden intermediate between non-carrier and control subjects; (3) that both PDV carriers and non-carriers have a stochastically greater burden of common risk variation than control subjects; and (4) that the effects of common and rare variants on liability for ASD likely combine additively. Regarding (3), it appears that ASD subjects carry a substantial burden of common risk variation, even if they also carry a rare PDV affecting risk. For (4), although common and rare risk variations likely act additively, the resolution imposed by the current data is coarse.

## Methods and results

### Data

Here, we present analyses of 17,972 samples of European descent (Additional file 1: Fig. S1): 3011 were diagnosed with ASD, while the remaining 14,961 were assumed to be unaffected. Among the ASD subjects, 1996 and 1015 came from SSC and PAGES, respectively. Control subjects came from the PAGES (1524) and eMERGE (13,437) cohorts. All subjects from the SSC and eMERGE were collected in the USA; subjects for PAGES come from Sweden. DNA from all subjects was genotyped on an Illumina genotyping platform: Human1M_v1, Human1M_Duov3, and HumanOmni-2.5 for SSC; Infinium OmniExpressExome-8 V1, Infinium OmniExpressExome-8 V1.1-V1.4, and Infinium Expanded Multi-Ethnic Genotyping Array for PAGES; and Illumina Human660W_Quad_v1_A for eMERGE (Additional file 1: Table S1). Genotypes for all samples were imputed at the Michigan Imputation Server [25] using the HRC reference panel [26]. Post-imputation quality control reduced the number of SNPs to 5,145,175. SNPs in high linkage disequilibrium (LD) were thinned using PLINK 2.0 [27] (--clump-r2 0.81; --clump-kb 50), producing 910,356 SNPs, and SNPs that were physically genotyped in more subsets were given preference in the thinning process. A final pruning step with parameters *r*^2^ < 0.64 for moving blocks of 50 SNPs and a window of 5 SNPs at a time reduced the genotyped dataset to 553,406 SNPs to be used for all GP calculations.

**Table 1:** Distribution of the subjects over the four ancestry clusters by ASD status and cohort

Cluster	ASD	Unaffected	Total	ASD		Unaffected	
				SSC	PAGES	PAGES	eMERGE
CL1	626	4034	4660	625	1	10	4024
CL2	873	5407	6380	813	60	74	5433
CL3	458	976	1434	372	86	165	811
CL4	1054	4444	5498	186	868	1275	3169
Total	3011	14,961	17,972	1996	1015	1524	13,437

### Genetic ancestry

To assess ancestry, 99,509 SNPs were selected because they were genotyped for all samples and were relatively independent (the larger set of SNPs was pruned to reduce linkage disequilibrium, as measured by pairwise *r*^2^: 50 SNP moving blocks, 5 SNP at a time, pairwise *r*^2^ < 0.64). Using genotypes from these SNPs, genetic ancestry was determined using function “clusterGem” in the package GemTools [28]. All 17,972 samples were clustered simultaneously using three ancestry eigenvectors, which divided the sample into four clusters (Table 1; Additional file S1: Fig. S2). Within each cluster, genetically matched pairs were chosen using the function “pairmatch” in library *optmatch* in R (1-to-1 fullmatch), which assessed pairwise distances among subjects based on the space defined by three ancestry eigenvectors (Additional file 1: Figs. S2–S3). The pairwise distances between the subjects of the matched pairs were far smaller than the average distance for all possible pairs (Additional file 1: Fig. S2).

### Approach to estimating G-BLUP

#### Overview

Our goal in this section is to employ a G-BLUP estimation procedure for GP that distinguishes ASD from unaffected subjects and is free of the influence of ancestry (see Additional file 1 for review of G-BLUP). Note that G-BLUP relies on genetically estimated relationships among subjects in a sample. To calculate this genomic relationship matrix (GRM), genotypes are standardized according to allele frequencies of the SNPs [29, 30]. Data from multiple subpopulations can be challenging if the number of subjects per subpopulation is unbalanced because allele frequency estimates are dominated by the largest subpopulation(s). As a result, the GRM is well estimated within the main subpopulation, but relationship estimates for subjects in other subpopulations will tend to be biased (Additional file 1: Tables S2–S3, Fig. S4). To overcome this concern, we divide our data into ancestry clusters; determine allele frequencies within clusters; standardize the genotypes within clusters; and then calculate the GRM based on these cluster-specific standardizations. This is not the sole concern regarding experimental design. If the ASD and unaffected subjects are not balanced over the ancestry space, bias can be induced in estimation of GP. Specifically, this imbalance can produce misleading differentiation between ASD and unaffected subjects. For this reason, we estimate GP based on a set of ancestry-matched case–control samples [31], thereby avoiding the problem of unbalanced sampling. See Supplemental Methods for a more detailed description of G-BLUP.

#### Genomic relationship matrix

Using unaffected subjects *not matched* to ASD subjects, we obtained within-cluster and overall allele frequency estimates. Then, from these two estimates, we used empirical Bayes methods, as described in Bodea et al. [32], to determine final cluster-specific allele-frequency estimates. These frequencies were then used to standardize cluster-specific genotypes and compute a cluster-specific GRM. We call this the cluster-specific GRM, or CLS-GRM, to differentiate it from a GRM computed from genotypes standardized by the mean allele frequency for each SNP using all 11,950 unmatched controls, which we call POP-GRM. Finally, we computed a GRM using the default approach implemented in GCTA software [29, 30], which uses all 17,972 samples (GCTA-GRM). We also used the GCTA software to calculate the first ten principal components of ancestry.

#### Approach to G-BLUP estimation of GP

For GP to discriminate ASD from unaffected subjects, the expected number of risk alleles in ASD subjects should be stochastically greater than that in unaffected subjects. As the difference in the relative number of risk alleles, which we call the “burden,” becomes greater between the two subpopulations, the GP becomes more accurate. A common way to build a GP is to split the data into a training set and a test set. For the training set, diagnosis is known and is used to develop the model for GP, which is then evaluated in the test set. A common breakdown is to use 90% of the sample for training, which can be done repeatedly using different portions of the sample. An expectation of statistical theory is that a larger training set yields a more accurate GP. For this reason, we chose a training and testing plan with N − 2 observations for training and a matched pair for testing. This is iterated over all matched possible pairs, making it also a computationally intensive plan. To make the plan feasible, we implemented computing techniques to expedite calculations (Additional file 1).

We analyzed *N* = 6022 (3011 matched pairs). CLS-GRM was used for the genetic relationship among the matched samples, without additional covariates. G-BLUP calculations require an estimate of heritability for ASD, which we set at 0.70 [33]. GP estimates were standardized to have mean = 0 and standard deviation = 1.

### Identifying carriers of PDVs

ASD subjects were classified as PDV carriers if their DNA had a protein truncating variant (PTV) or deleterious missense variant (missense badness, PolyPhen-2, and constraint score, MPC > 2) [34] in one of 102 genes identified in Satterstrom et al. [13] as affecting risk. DNA from ASD subjects from the SSC sample was characterized for de novo PDVs using whole-genome sequence, as reported by An et al. [35]. DNA from PAGES affected subjects was characterized for PTV and MIS variants from whole-exome sequence, as reported in Satterstrom et al. [13]: 778 out of 1015 ASD subjects were analyzed and de novo status was unknown for all PDVs. For the PAGES subjects whose DNA was not characterized, we assumed they were non-carriers.

We also included CNVs as PDVs. For the PAGES sample, we used the set of damaging CNVs described in Mahjani et al. [36], who identified CNVs for 956 out of the 1015 ASD subjects we analyzed. For the SSC sample, CNVs were identified by Sanders et al. [14] and damaging status defined following Mahjani et al. [36]. For each dataset, subjects who carried a trisomy or had large or multiple CNVs were set to “undetermined” for carrier status and thus were not in the PDV carrier versus non-carrier analyses, although they were retained as ASD subjects.

DNA from 305 ASD subjects carried one or more PDVs (Table 2; Additional file 1: Table S4). If a subject carried multiple PDVs, the most severe PDV for each subject was counted, under the assumption that the ranking of severity was CNV > PTV > MIS [14].

**Table 2:** Counts of most severe potentially damaging variants (CNV > PTV > MIS)

	Total	SSC	PAGES
CNV	166	78	88
PTV	78	58	20
MIS	61	38	23
Non-carrier	2682	1814	868

### Results for GP

**Fig. 1:**
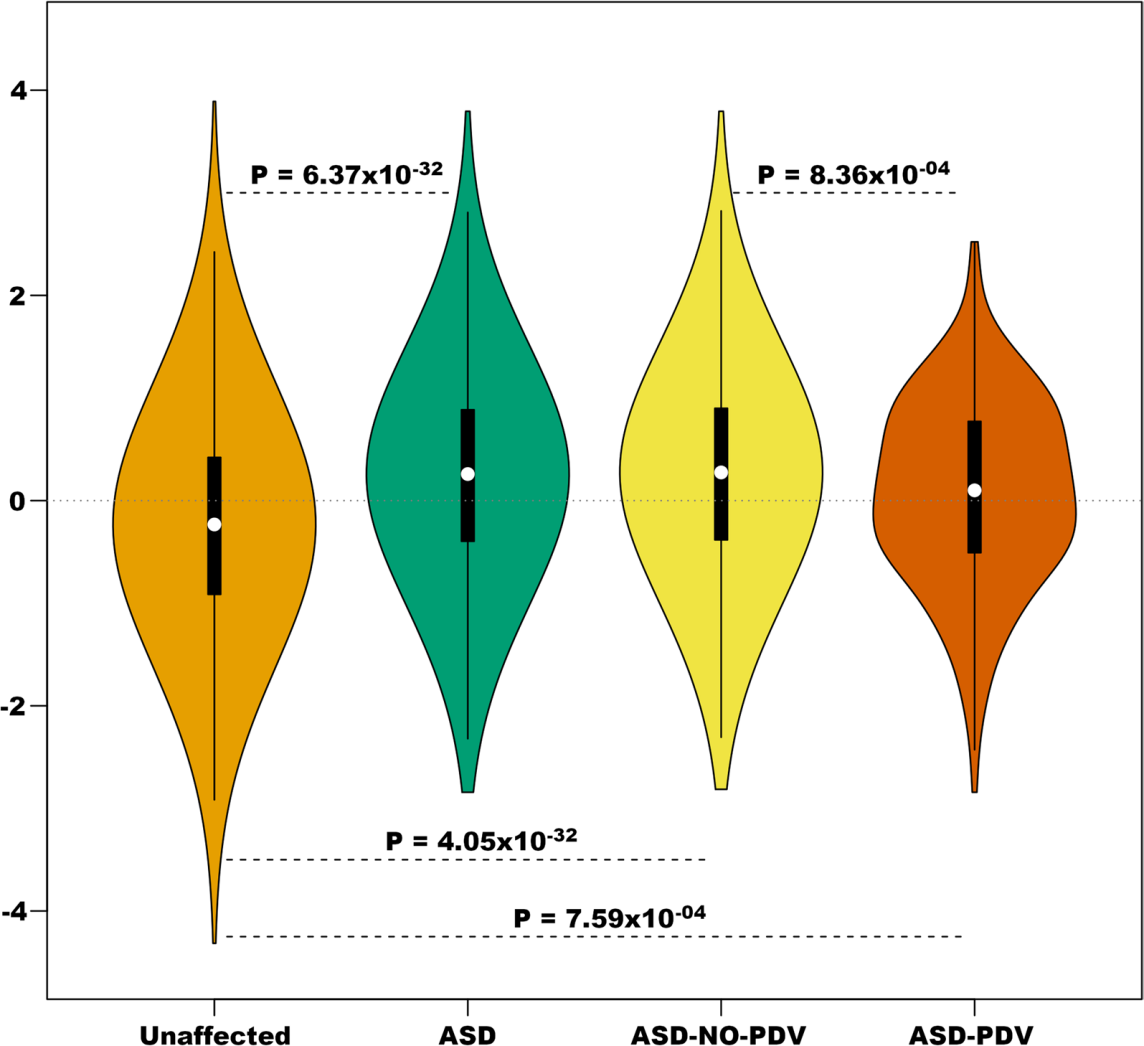
The burden of risk variation by ASD status and by carrier status of ASD subjects. Burden is estimated using GP, standardized to have mean = 0 and standard deviation = 1. *ASD-NO-PDV* ASD subjects with no known potentially damaging variants, *ASD-PDV* ASD subjects with known potentially damaging variants

To ensure matching was adequate, we first tested whether GP differed between clusters, after controlling for diagnosis. In this analysis-of-variance model, GP did not differ significantly by cluster (*F* = 0.795, *df* = 3, 6017; *P* = 0.497). The relative risk of being an ASD subject, as opposed to unaffected, increased with GP (logistic regression OR = 1.67; 95% CI 1.58–1.77; *P* = 6.73 × 10^−32^; pseudo-*R*^2^ = 7.80%). Because GP is continuous and standardized, the increased risk should be interpreted in units of standard deviations of GP. Within ASD subjects, PDV carriers had a significantly smaller burden of common risk variants, on average, than do non-carriers (Fig. 1), which is also reflected in the relative risk as a function of GP (OR = 0.81; 95% CI 0.71–0.92; *P* = 8.36 × 10^−4^). Both PDV carriers and non-carrier ASD subjects had a greater average GP than unaffected subjects (Fig. 1). Including three or ten eigenvectors of “ancestry” as covariates in the model did not alter these conclusions (Additional file 1: Tables S5–S6), although adding ten eigenvectors as covariates diminished somewhat the difference in GP between ASD and unaffected subjects. One possible explanation is that eigenvectors can be a function of both the burden of risk variation and ancestry.

Conducting other exploratory analyses, we showed the following: CLS allele frequencies were better for standardizing genotypes than population-level allele frequencies (Additional file 1: Table S7); in some settings, adjusting by eigenvectors of ancestry can overcome the inaccuracy induced by population-level allele frequencies (Additional file 1: Table S8); our training/testing plan of N-2/2, where the two individuals comprise a matched pair, was optimal relative to other N-X/X for X > 2 (Additional file 1: Tables S9–S10), and for data sets imbalanced in affected and unaffected subjects within the genetic ancestry space, such as Table 1, this imbalance biased GP (Additional file 1: Fig. S5). We note that while the training/testing plan of N-2/2 appears optimal, it is also over-fitting the data. Therefore, all significance tests contrasting ASD versus unaffected GP were corrected by genomic control (GC, *λ* = 2.406) [37]; see Additional file 1: Fig. S6. Estimating GP by cohort shows that both contribute to mean differences between ASD and unaffected individuals (Additional file 1: Table S11). GP is less accurate for the PAGES than for the SSC cohorts, which is consistent with several factors: Ascertainment of the PAGES and SSC cohorts was quite different; PAGES ASD subjects were more likely to be carriers, as judged by possibly damaging CNVs (Table 2); and sample size was larger for the SSC than PAGES cohorts and GP will tend to be tailored to the attributes of the larger sample.

### Polygenic risk scores and a weighted score

#### Approach

We evaluated two PRS, specifically one derived from an ASD GWAS [6] and the other from a recent schizophrenia (SCZ) GWAS [20] using a pruning and thresholding approach (Additional file 1), with the threshold set to include only SNPs with *P* values < 0.01. As described in “Background”, this threshold is chosen to ensure the PRS would be relatively independent of GP. For the ASD GWAS, we used the results based only on the Danish iPsych data because SSC and a portion of the PAGES data were included in the full GWAS analysis [6]. After quality control (QC), described in Additional file 1 9,983 and 26,972 SNPs were included for ASD and SCZ PRS, respectively.

#### Results for PRS

**Fig. 2:**
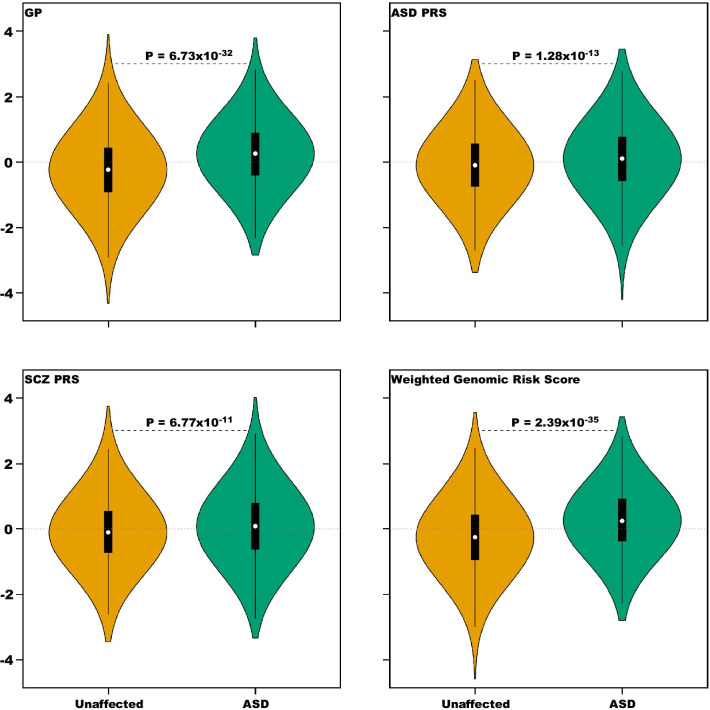
Distribution of risk scores divided by ASD and unaffected subjects. Burden is estimated using GP, an ASD polygenic risk score (ASD PRS), a schizophrenia polygenic risk score (SCZ PRS), and a weighted genomic risk score that incorporates information from GP, ASD PRS, and SCZ PRS

**Fig. 3:**
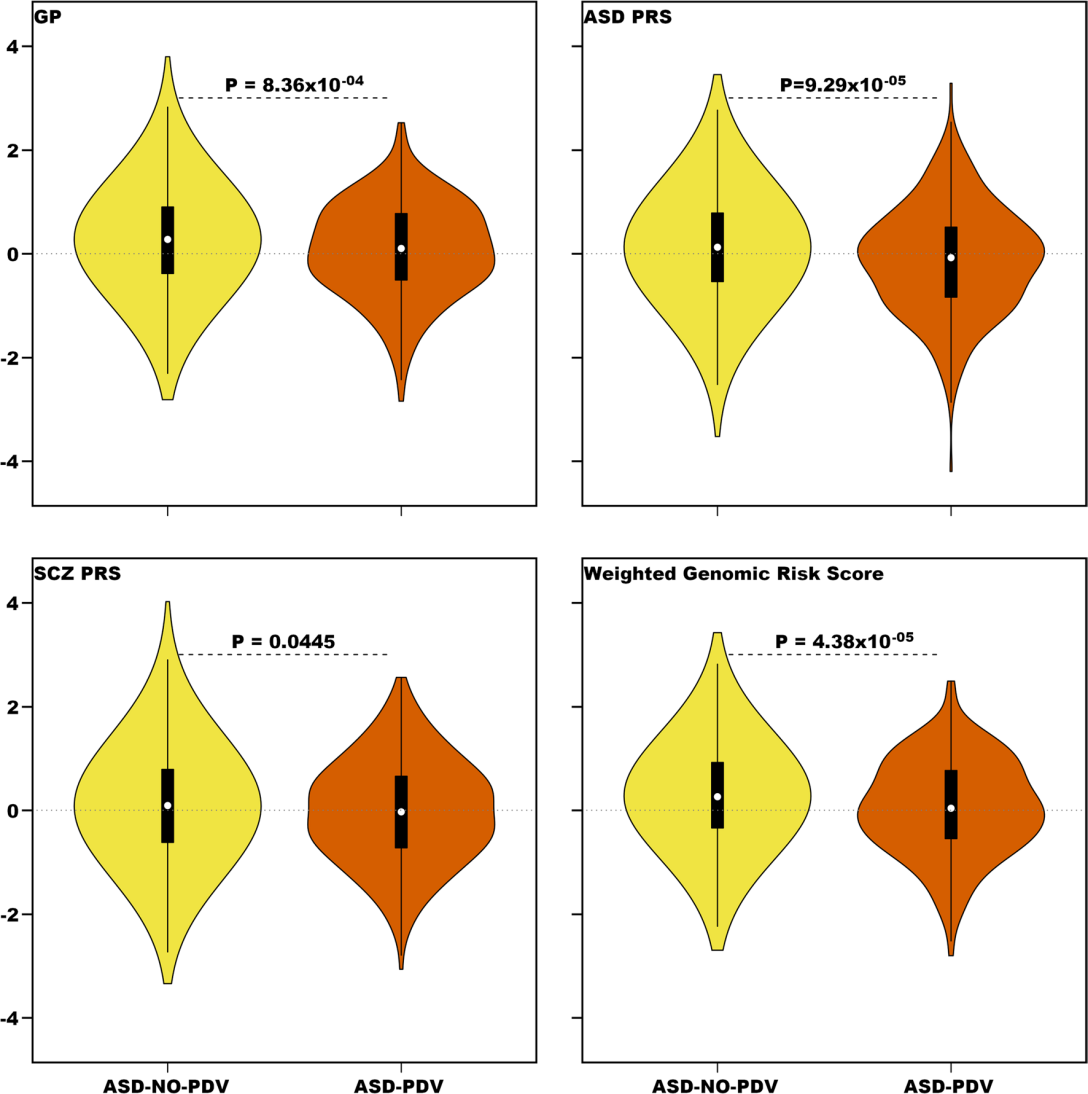
Distribution of risk scores divided by ASD PDV carriers and non-carriers. Burden is estimated as noted in Fig. 2, here comparing ASD PDVs carriers (ASD-PDV) to non-carriers (ASD-NO-PDV)

For the 3011 matched pairs previously described, both the ASD-PRS and the SCZ-PRS distinguished ASD from unaffected subjects, on average (Table 3; Fig. 2). The ASD-PRS also was significantly different for PDV carrier versus non-carrier ASD subjects, as is the SCZ-PRS (Table 3; Fig. 3). Results for a pTDT analysis for ASD were similar to those for the PRS (Additional file 1: Table S11). We also explored a PRS built from a GWAS for educational attainment: it weakly distinguished ASD from unaffected subjects, but not carrier versus non-carrier status (Additional file 1).

**Table 3:** Logistic regression of ASD status on G-BLUP and PDV status in cases on G-BLUP, ASD-PRS, SCZ-PRS, and the weighted genomic risk score (WGRS)

Score	ASD status				PDV status (cases only)		
	OR	95% CI	*P*	Pseudo-*R*^2^ (%)	OR	95% CI	*P*
G-BLUP	1.67	1.58–1.77	6.73 × 10^−32^	7.80	0.81	0.71–0.92	8.36 × 10^−4^
ASD-PRS	1.21	1.15–1.28	1.28 × 10^−13^	1.23	0.79	0.70–0.89	9.29 × 10^−5^
SCZ-PRS	1.19	1.13–1.25	6.77 × 10^−11^	0.95	0.89	0.79–1.00	0.0445
WGRS	1.73	1.64–1.83	2.39 × 10^−35^	8.74	0.77	0.68–0.87	4.38 × 10^−5^

#### Combining scores

The correlation between GP and ASD-PRS was 0.079 (*P* = 9.80 × 10^−10^), while it was 0.099 for GP and SCZ-PRS (*P* = 1.16 × 10^−14^). ASD-PRS and SCZ-PRS were stochastically uncorrelated (*r* = 0.007, *P* = 0.581). Thus, given their modest correlations, these three scores had essentially independent information. To combine them into one weighted genomic risk score (WGRS), we based the weights on their Nagelkerke’s pseudo-*R*^2^, which roughly measures each score’s ability to separate ASD and unaffected subjects (Table 3). The WGRS was standardized to have mean 0 and standard deviation of 1. Compared to the other single scores, WGRS better distinguished ASD from unaffected subjects and carrier/non-carrier status of ASD subjects (Table 3; Figs. 2, 3).

We also explored two alternative weighting schemes for obtaining a WGRS, compared to our a priori approach: 1) equal weights and 2) an optimal weighting scheme based on training/testing of the data, as described in Additional file 1: Table S11. Both led to similar WGRS, compared to our a priori approach, and thus also produced similar results.

### Relationship between common and rare risk variants

**Fig. 4:**
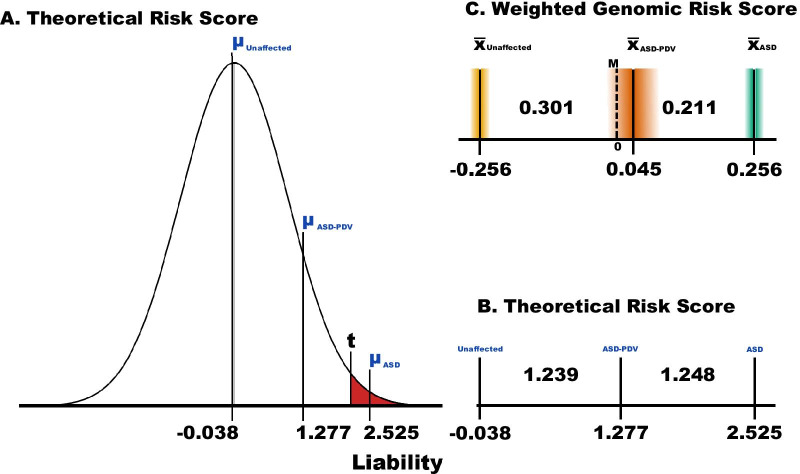
Continuous liability model for ASD compared to empirical realization. **a** Liability is assumed to be normally distributed in the population, and there exists a threshold t of liability beyond which everyone is affected and below which no one is affected. ASD prevalence determines t and the average risk of ASD and unaffected subjects; given an estimate of the relative risk of ASD due to rare damaging variants, the average risk of PDV carriers of these PDVs is also specified. **b** A portion of A, highlighting the relatively even spacing between its average risks. **c** The realized average risk of these three groups, as measured by the weighted genomic risk score with their 95% confidence intervals (Fig. 3)

We next ask about the interplay of common and rare risk variants. While diagnosis of ASD is binary, a person either does or does not meet diagnostic criteria, continuous variability of phenotypes related to ASD has long been recognized. A related mathematical observation is that a continuous liability model, in which a normally distributed liability is determined by genetic and environmental risk factors carried by each subject in a population, can be an excellent model for a binary trait like ASD (Fig. 4). In such a model, a liability threshold *t* determines whether an individual meets the diagnostic criteria and this threshold maps onto ASD prevalence (Fig. 4). If we take prevalence to be 1.5% for the population [13], it sets the threshold for diagnosis of ASD, in terms of a z-score, establishes the mean liability for ASD and control subjects, and thus defines the difference in average liability between ASD and unaffected subjects (Fig. 4).

For this prevalence, the average liability for ASD subjects is 2.525 and for unaffected subjects it is slightly below zero, − 0.038 (Fig. 4a). To determine where the average carrier ASD subject would fall on this continuum of liability, we require an estimate of the relative risk due to such PDVs. Using results from Satterstrom et al. [13] and Sanders et al. [14], a relative risk of 15 is a good approximation. We can use standard theory, which is described in Satterstrom et al. [13], and elsewhere, to compute the expected liabilities for unaffected and affected individuals, as well as PDV carrier and non-carrier ASD status, assuming common variants and PDVs combine additively to determine liability. Liability for the average carrier would be 1.277 (Fig. 4a), which falls close to the mid-point between the average liabilities of ASD and unaffected subjects, 1.282 (Fig. 4a, b). How does this compare to results for WGRS (Fig. 4c)? For ASD PDV carriers, the average WGRS is 0.045, close to the midpoint (0.0) between the average WGRS for ASD (0.256) and unaffected subjects (− 0.256). Thus, these calculations suggest that rare damaging risk variants and common risk variation act roughly additively to confer liability to ASD. Moreover, when we evaluated whether these results could simply arise by the way we estimated GP, they could not (Additional file 1).

**Fig. 5:**
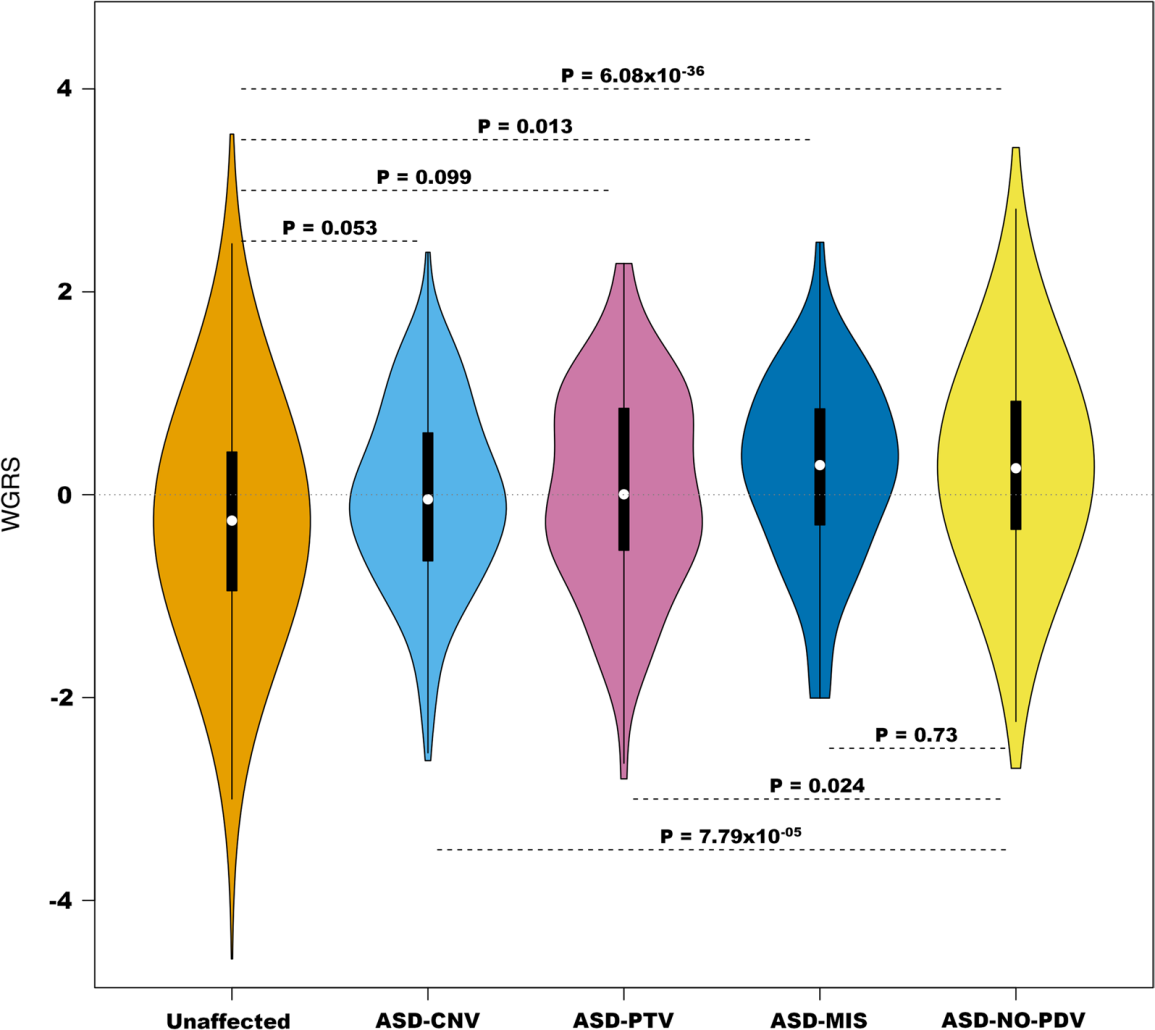
Distribution of risk scores for different subsets of subjects. Unaffected, ASD PDV carriers with copy number variants (ASD-CNV), loss of function variants (ASD-PTV), and missense variants (ASD-MIS) and ASD non-carriers (ASD-NO-PDV)

The number of ASD PDV carriers is too few to evaluate liability much more deeply, at least reliably. If the additive model were a close approximation to reality, we would anticipate that the burden observed in ASD subjects would vary inversely with the relative risk of ASD associated with the damaging rare variants they carry. Estimated relative risks associated with pathogenic CNVs and PTV tend to be similar and large [14], whereas the relative risk associated with missense PDVs tend to be far smaller. When we partition PDV carriers according to the type of variant they harbor and compare their burden to that of non-carriers and unaffected subjects, results are consistent with an additive model (Fig. 5): The average burden for ASD subjects carrying CNVs is smallest, although only slightly smaller than the average burden of those carrying PTV PDVs; as might be expected, the difference between these two groups is not meaningful; whereas the average burden of ASD subjects carrying missense variants is substantially larger. By contrast, one might expect that carriers with PDVs in genes commonly disrupted in ASD subjects, such as *CHD8* [38–40], would bear a smaller burden, on average, than carriers of PDVs in genes disrupted far less often. A confounder here is gene size, larger ASD risk genes will tend accrue more PDVs than smaller genes. While imperfect, a rough way to account for this confounder is to use the TADA Bayes factor [41], which summarizes the evidence for association from the expected spectrum of PDVs, based on gene size and nucleotide content, to that observed in the sample. Comparing this Bayes factor, we used log_10_(BF) to account for the vast range of BF values, for the 102 inferred ASD genes identified in Satterstrom et al. [13] to the burden harbored by ASD subjects carrying PDVs in those genes, we do not find a significant relationship with log_10_(BF) (*b* =  − 0.015; *P* = 0.214), although the relationship is negative, as expected.

## Discussion

Here, we asked how rare and common risk variation jointly affect liability for ASD. We analyzed two samples characterized for both types of variation. Based on genotypes of common variation, we computed a small set of risk scores, each of which is likely to describe a portion of the genetic risk of ASD attributable to common variation. We also computed a weighted average of these scores, WGRS, which tended to perform better than any single score at differentiating ASD and unaffected subjects (Fig. 3) and at differentiating ASD subjects who carried rare PDVs likely to affect risk—PDV carriers (Fig. 5)—from ASD subject who were not known to carry such variants (non-carriers). By contrasting patterns of the expected and observed burden of common risk variation in PDV carriers and non-carriers (Fig. 4), we conclude that the preponderance of evidence suggests that rare and common risk variation combine additively in their effects on ASD liability. This agrees with conclusions from other researchers [7, 10, 42].

It is worthwhile emphasizing, however, that the evidence presented here is far from conclusive and it differs from that of other studies. Consider the study by Weiner and colleagues [7], which includes many of the authors of this current manuscript as collaborators. It introduces the pTDT, which uses three key pieces of information to evaluate association: a previously established PRS function; the average of the PRS of mother and father, the mid-parent average; and the deviation of the offspring from the mid-parent average. Using this information, they show that the pTDT is an effective tool for genetically discriminating ASD probands from their unaffected siblings. Moreover, they establish that both carriers and non-carriers, as groups, carry a stochastically greater burden of common risk variants relative to unaffected siblings. Yet, carriers have a pTDT score of 0.17, on average, somewhat but not significantly greater than the average score for non-carriers, 0.12 (their Additional file 1: Table S13). Analyzing a broader set of developmental disabilities, Niemi and colleagues [42] report similar findings to those of Weiner and colleagues [7], specifically carriers have genetic scores indistinguishable from non-carriers and both carry greater burden of risk variation. Both studies use the pTDT approach, and both conclude that rare and common variations combine additively to affect risk.

In contrast to those results [7, 42], in our study the average burden for carriers falls between that for unaffected and non-carrier affected individuals and this we view as evidence for additive effects. The conclusions of our studies are not completely at odds, although they do not fit perfectly together either. That the burden of common risk variants is greater in carriers and non-carriers in all three studies, relative to expectation, is consistent with common variation contributing to liability. What remains unresolved is how it combines with rare variants if they also have a large impact on liability. If PDVs found in individuals with ASD or severe developmental disability were close to completely penetrant—thus having a large impact on liability—then little or no contribution from common variation would be necessary for a diagnosis. Under this model, effects of PDVs are sufficient to cause developmental disability [42] or ASD [7]; common variation would induce variation about the mean liability for affected individuals and perhaps alter presentation of the phenotype. This is observed for quantitative phenotypes of other rare genetic disorders [10]. However, in this scenario the expected liability arising from common variation in carriers should be near zero, as opposed to the significant positive estimates found by Weiner [7] and Niemi [42]. What explains the excess of common variation found in the carriers from their studies? One reasonable possibility is that stochastic variation plays a complicating role. If some PDVs were of sufficient impact on liability to cause the ASD or other developmental phenotype, whereas others were not, and if the impact of this latter group on liability combined additively with common variation, then the fraction of each type of PDV would determine where the mean liability of carrier subjects fell on the continuum between unaffected and non-carrier subjects. Such a model would induce greater variability in the average score for carriers, perhaps sufficiently to make the average burden estimated from carriers and non-carriers indistinguishable.

With larger samples than presented here, more compelling evidence could be drawn from an evaluation of carriers of PDVs in genes with very different recurrence rates in ASD individuals. For example, certain genes, such as *CHD8* [38–40], are often found to carry PDVs in ASD individuals. Other genes show significant association, yet far less recurrence. In future studies and with a much larger sample, we should be able to order ASD risk genes accurately in terms of the relative risk of ASD generated by PDVs in these genes and evaluate how common variant risk changes along this ranking. If the two sources of risk work additively, they should show a strong negative relationship.

Why do we need to evaluate the nature of the relationship between common and rare variations so thoroughly? Suppose, for example, that some of the ASC’s 102 ASD genes do not truly affect risk and half of the *assumed* PDV carriers have PDVs in these genes. Under this unlikely but not impossible scenario, these subjects would, in expectation, carry the mean WGRS observed in non-carriers (Fig. 4). To achieve the mean WGRS observed for the entire population of *assumed* PDV carriers, which consists of an equal mixture of true carriers of risk PDVs and non-carriers, the mean for the subpopulation of true carriers would, in expectation, fall at the mean for unaffected individuals (Fig. 4). Under this scenario, joint effects of rare and common risk variants are irrelevant, a rare PDV would always be sufficient to cause ASD. Such scenarios can only be completely ruled out by using alternative ways of evaluating whether rare and common risk variation combine additively in their effects on ASD liability.

If common variant risk burden of PDV carriers is substantial, as the results here suggest, they have implications for genetic counseling regarding recurrence risk of ASD. Currently, genetic counseling for recurrence risk is binary, depending on whether or not a rare PDV in an ASD gene is found in the proband’s genome. If such a PDV is found, then the PDV is typically assumed “causal” for the proband’s ASD and recurrence probability for ASD is its prevalence. When this assumption is a good approximation, and it will be for many PDV carriers (9], counseling is also a good approximation. In some families, however, the PDV carrier has ASD in large part because of the polygenic burden carried by the parents and in this instance the current advice for recurrence risk is inaccurate. To give a concrete example, when we examined loss-of-function carriers in the SSC, we estimated that over 40% of these individuals would still have ASD even without the loss-of-function PDV [9], and this would be predicted to be even more of an issue with less penetrant variation (e.g., missense variation). Because the present state of knowledge does not allow us to know, a priori, which scenario is relevant, it is important for genetic counselors to consider this uncertainty and whether it should be built into their advice for parents regarding recurrence risk.

## Limitations

An ideal study would have even larger sample size than the one we present here. Given the incomplete knowledge of genes and PDVs in ASD, a small fraction of subjects that are categorized as non-carriers could be PDV carriers. In addition, not all DNA from PAGES ASD subjects was characterized for rare sequence variants through exome sequencing and they were assumed to be non-carriers because the vast majority would be. Inherent in the name PDV, we cannot be certain all of these variants actually carry risk of ASD. Indeed, in final review an editor asked for a set of potentially damaging CNVs (pdCNVs) to be re-evaluated for this reason. While taking away these pdCNVs, which removes 28 ASD PDV carriers from our analyses, had little impact on our results (Additional file 1: Table S14), it underscores this uncertainty as a limitation to our analyses. Finally, it is possible that some of the individuals participating in the eMERGE study, who we assumed were unaffected, could be affected (see [43]). These drawbacks limit our ability to go beyond coarse characterization of interplay of rare and common variations.

## Conclusion

While rare and common variations confer liability for ASD, how they jointly confer liability is an open question. By analyzing data from 3011 affected subjects and 3011 genetically matched unaffected subjects, we conclude that the burden of common risk variants borne by ASD subjects is stochastically greater than that borne by control subjects and that ASD subjects who carry rare potentially damaging variation conferring risk of ASD have an average burden intermediate between non-carrier ASD and control subjects. The effects of common and rare variants likely combine additively to determine individual-level liability.

## Supplementary Information

**Additional file 1.** Supplementary information for joint effects of rare and common variation.

## Data Availability

All results from analyses are presented in the manuscript and Additional file 1.

## Abbreviations

ASD: Autism spectrum disorder
ASD-NO-PDV: Non-carrier ASD case
ASD-PDV: PDV carrier ASD case
CLS: Cluster-specific
CNV: Copy number variant
eMERGE: Electronic MEdical Records and Genomics Network
G-BLUP: Genomic-Best Linear Unbiased Prediction
GCTA: Genome-wide complex trait analysis
GP: Genomic prediction
GRM: Genomic relationship matrix
GWAS: Genome-wide association studies
HRC: Haplotype Reference Consortium
LD: Linkage disequilibrium
MIS: De novo missense variant
PAGES: Population-Based Autism Genetics and Environment Study
pdCNV: Potentially damaging CNV
PDV: Potentially damaging variant
POP: Population
PRS: Polygenic risk score
pTDT: Polygenic transmission disequilibrium test
PTV: Protein truncating variant
QC: Quality control
SSC: Simons Simplex Collection
SCZ: Schizophrenia
TDT: Transmission disequilibrium test
WGRS: Weighted genomic risk score
