# Supplementary material for: How rare and common risk variation jointly affect liability for autism spectrum disorder
Source: Mol Autism. 2021 Oct 6;12:66. doi: 10.1186/s13229-021-00466-2 (PMC8495987; doi:10.1186/s13229-021-00466-2)
Supplement: Supplementary file 1 — Additional file 1. Supplementary information for joint effects of rare and common variation. [file 13229_2021_466_MOESM1_ESM.docx]

**ADDITIONAL FILE 1**

**G-BLUP.**

For a detailed description of the set-up of G-BLUP, see[1]. G-BLUP is based on mixed model methodology in which the ASD outcome for individual (y_i_) is modeled as a function of fixed covariates (mean and ancestry, β_i_) and a random genetic effect (u_i_) and a random residual (e_i_). In matrix form this becomes:

$$y=X\beta+Zu+e$$

Let n be the number of observations, cases plus controls; and p be the number of covariates, then

y, u, and e are vectors of length n; β is a vector of length p; X is a matrix of size n×p connecting the covariate effects to the observations; and Z is a “picker” matrix with 0/1 entries of size n×n tying the random genetic effect to the right observation y. It is assumed that $\left( \begin{matrix} y \\ u \\ e \end{matrix} \right)\sim MVN\left( \left( \begin{matrix} X\beta\\ 0 \\ 0 \end{matrix} \right),\left( \begin{matrix} ZGZ^{'}+R & GZ' & R \\ ZG & G & 0 \\ R & 0 & R \end{matrix} \right) \right)$, where G is the genetic covariance among the individuals $G=\Gamma\sigma_{g}^{2}$ with Γ the estimated relationships among the samples and $\sigma_{g}^{2}$ an estimate for the genetic variance. Here we use the genomic relationship matrix estimated from our genotype data for Γ. In addition, $R=I\sigma_{e}^{2}$ the covariance among the independent residuals and $\sigma_{e}^{2}$ is an estimate of the residual variance.

To obtain solutions for these equations under the stated assumption one can use the mixed model equations (MME) as derived by Henderson[2]:

$$\left( \begin{matrix} X'X & X'Z \\ Z'X & Z^{'}Z+\Gamma^{-1}\alpha\end{matrix} \right)\left( \begin{matrix} \beta\\ u \end{matrix} \right)=\left( \begin{matrix} X'y \\ Z'y \end{matrix} \right)$$

where $\alpha=\frac{\sigma_{e}^{2}}{\sigma_{g}^{2}}=\frac{1-h^{2}}{h^{2}}$ (using $h^{2}=\frac{\sigma_{g}^{2}}{\sigma_{g}^{2}+\sigma_{e}^{2}}$). For our analysis we set h^2^ = 0.70. Admittedly, y is not normally distributed, but the underlying liability distribution can be assumed to be.

While the equations above will determine the genetic effect for each individual with a phenotypic observation, the interest here is in predicting the risk attributable to the genetic make-up of a sample for which we do not know the ASD status, which we call GP (although we could also call it G-BLUP). Instead of having Γ made up of only samples with known ASD status this can be expanded to include samples for which there are genotypes but no ASD information. Solving the MME will then give GP for the individuals without ASD status.

When estimating $u_{j}$ using MME, we combine information on the ASD status of the sample of interest with information on the ASD status of all other samples based on the genetic relationships among the samples. For phenotypes with high heritability, this will be driven by the sample’s own ASD status. For GP we want this estimate to be free of the sample’s own status and we want our estimate to solely rely on the ASD status of genetically related samples. E.g., if we do not know the ASD status of a sample what would be our best prediction of their ASD burden? The easiest way to accomplish this is to pretend that the ASD status of sample j is not known, adjust the MME accordingly, and then solve the MME including sample j. When using matched pairs, we set both the ASD status of the affected and the unaffected individuals of the matched pair to unknown and solve the MME for the pair. We repeat this for each matched pair. This will require obtaining solutions for the complete set of MME for each matched pair.

To expedite calculations, we used the following approach. Let Ax=b represent the MME based on not knowing the ASD status of the matched pair of interest that we need to solve. We first re-arrange A and b such that the matched pair of interest are in the last two rows and columns of A and the last two elements of b and y. We can then solve for this pair as follows. Let the Cholesky decomposition of A be L and A=LL’, in which L is a lower triangular matrix. Now, to solve Ax=b, first solve Lz=b where z=L’x. This is the forward step. Because of the special structure of L, these solutions can be obtained using row-wise elimination avoiding calculation of the inverse of L. Once z has been obtained, solve L’x=z to obtain the solutions x using the elimination in reverse, the backward step. Because we are only interested in the solutions for the last two equations, the pair whose GP are to be predicted, the backward step only involves solving the last two equations, which we do through backwards elimination.

**Genotyping Arrays.**

**Additional File 1: Table 1**. Distribution of subject DNA characterized by Illumina microarrays. Here we present the total ASD and unaffected individuals of European ancestry included in our analyses as well as the 3,011 matched pairs, as described in the main manuscript and below.

|  |  | Total Counts | | Paired Count | |
| --- | --- | --- | --- | --- | --- |
| Dataset | Platform | Unaffected | ASD | Unaffected | ASD |
| SSC | 1Mv1 | 0 | 271 | 0 | 271 |
| SSC | 1Mv3 | 0 | 920 | 0 | 920 |
| SSC | Omni2.5 | 0 | 805 | 0 | 805 |
| PAGES | MEGAEX^1^ | 1,295 | 0 | 811 | 0 |
| PAGES | Omni_v1 | 109 | 218 | 32 | 218 |
| PAGES | Omni_v1_1 | 0 | 121 | 0 | 121 |
| PAGES | Omni_v1_2 | 120 | 491 | 39 | 491 |
| PAGES | Omni_v1_4 | 0 | 185 | 0 | 185 |
| EMERGE | Human_660W | 13,437 | 0 | 2,129 | 0 |
|  |  | 14,961 | 3,011 | 3,011 | 3,011 |

^1^MEGAEX: Multi-Ethnic Genotyping Array, Expanded.

**Identification of European Ancestry.**

Ancestries for the SSC subjects were determined previously[3]. To identify subjects of European ancestry from PAGES and EMERGE, we evaluated their genetic ancestry within the context of the data from subjects drawn from three 1000 Genomes (1000G) population samples: CEU (European), YRI (Yoruba), and CHB (Chinese). We then chose the subjects to be of European decent if their estimated ancestry representation mapped near CEU using GemTools (results not shown). Using all subjects selected to be of European ancestry, including SSC, PAGES and EMERGE, we next projected genetic ancestry by GemTools onto the ancestry space including the same three 1000G populations as described above (Fig. 1). Additionally, to show the similarities and differences of the spectral embedding derived from the normalized Laplacian of a graph[4, 5], as implemented in GemTools, with the more standard approach of PCA, we used GCTA to implement the PCA ancestry analysis (Fig. 1). In the leading two dimensions, the spectral decomposition recognizes the north-south gradient in Europeans, as well as other differences, whereas those features emerge in later dimensions for PCA. In those later dimensions (PCA3/4), the PCA analysis merges Han Chinese with Europeans whereas spectral decomposition does not. If the goal is to identify subjects of European ancestry from genotypes, however, either approach is effective.

To account for variation within European ancestry, subjects were clustered using GemTools and three ancestry eigenvectors to identify four clusters of subjects (Fig. 2). Then, within each cluster, genetically matched pairs were chosen using the function 'pairmatch' in R library optmatch (1-to-1 fullmatch), which assessed pairwise distances among subjects based on the space defined by the three ancestry eigenvectors. Because key analyses were performed on matched pairs of subjects of European ancestry, we next ensured that all those pairs also map onto European ancestry as laid out in Fig.1 (Fig. 3).

**Additional File 1: Figure 1**. Ancestry space created based on the 17,972 samples used in this study projected into which CEU (European), YRI (Yoruba), and CHB (Chinese) were also added. Panels A and C show results from spectral decomposition, while B and D show results from principal component analysis.


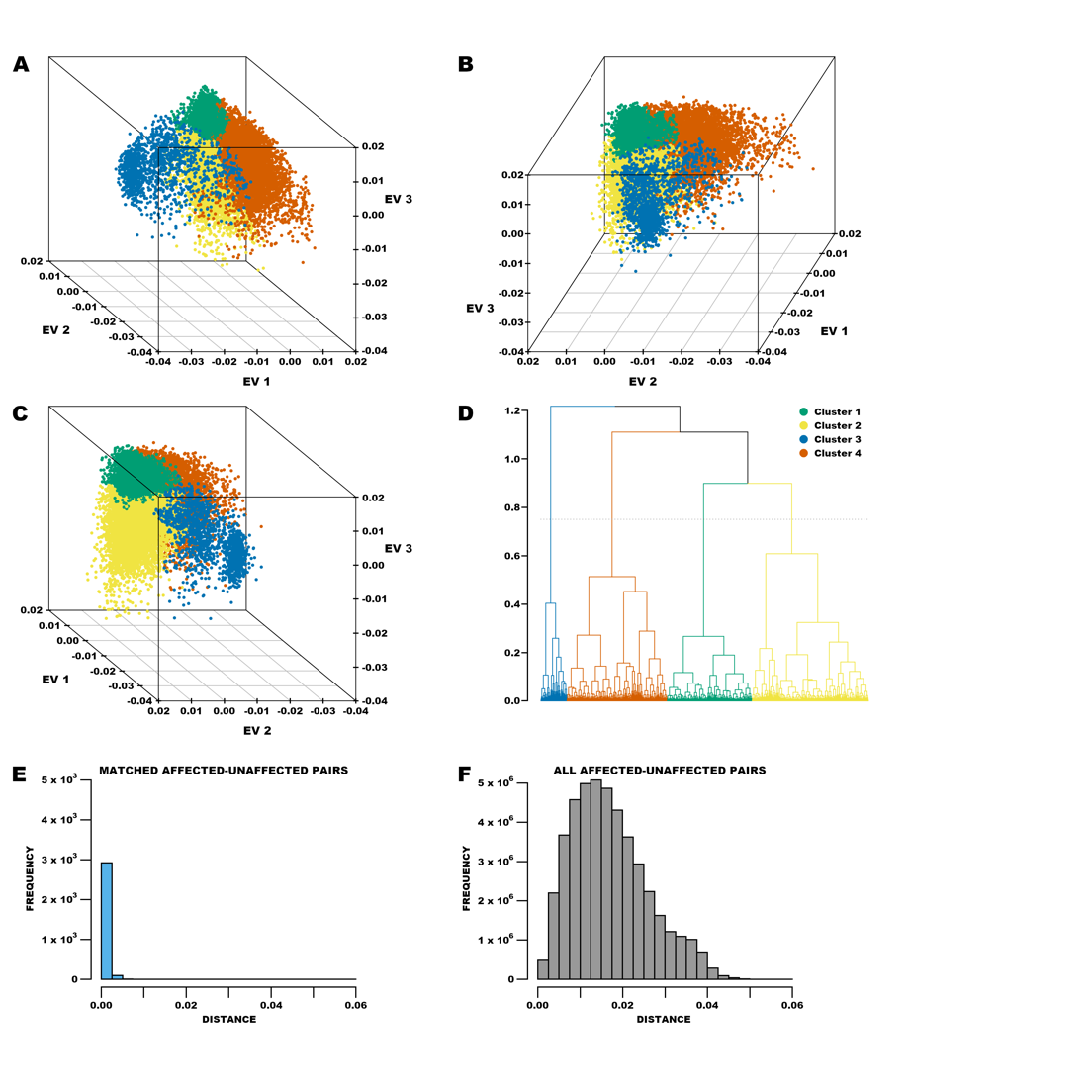


**Additional File 1: Figure 2**. Clustering of the 17,972 samples to control for variation in Europeans. A-C: different views of the 3D ancestry space; D: hierarchical cluster. E-F: distance between 3,011 matched pairs (E) and all possible pairs (F) in the ancestry space. The ancestry space was created using GemTools without the use of 1000G samples.

**Additional File 1: Figure 3**. Location of the 3,011 matched pairs in the ancestry space created using all 17,972 samples and to which the 1000G subjects from CEU, YRI, and CHB were added. Panels A and C show results from spectral decomposition, while B and D show results from principal component analysis.

**Relationships Among Samples.**

Estimated relationships amongst pairs of samples are influenced by the allele frequencies used to standardize their genotypes. If poorly chosen allele frequencies are used for this standardization, it can lead to an apparent excess of homozygosity of subjects and an increased level of relationships among subjects. A common situation when this will occur is the mixture of population samples of different ancestry. Because G-BLUP analyses are a function of the estimated relationships among subjects, ignoring the structure can bias its estimates. To evaluate this issue, we assessed a measure of homozygosity within the four ancestry clusters of subjects we analyzed for GP, as well as the estimated relationships among the subjects within cluster. For this analysis, we evaluated results using either the cluster-specific allele frequencies or the global allele frequencies as used in GCTA (Tables 2-3, Fig. 4). Notably, estimates for homozygosity are significantly inflated in some clusters when the Global allele frequencies are used, as is the variance in relationships (as we would expect if some pairs of subjects are estimated to be more closely related). Both of these effects are expected if ancestral variation among Europeans were not taken into account. Another approach we could have taken is to control for this ancestry variation using principal components of genetic ancestry and this too will be effective in many settings.

**Additional File 1. Table 2**. Variation in homozygosity estimates using within cluster allele frequencies versus global frequency estimates.

| Cluster | Within Cluster | Global | Ratio | p-value^1^ |
| --- | --- | --- | --- | --- |
| CL1 | 2.99×10^-5^ | 2.90×10^-5^ | 1.031 | 0.593 |
| CL2 | 4.05×10^-5^ | 3.85×10^-5^ | 1.051 | 0.294 |
| CL3 | 2.62×10^-4^ | 4.86×10^-4^ | 0.541 | 3.29×10^-20^ |
| CL4 | 4.43×10^-5^ | 4.52×10^-5^ | 0.981 | 0.657 |

^1^Variance ratio test testing deviation from 1.

**Additional File 1. Table 3**. Variation in relationship estimates using within cluster allele frequencies versus global frequency estimates.

| Cluster | Within Cluster | Global | Ratio | p-value^1^ |
| --- | --- | --- | --- | --- |
| CL1 | 1.08×10^-5^ | 1.08×10^-5^ | 1.000 | 0.917 |
| CL2 | 1.08×10^-5^ | 1.08×10^-5^ | 1.003 | 0.917 |
| CL3 | 2.71×10^-4^ | 6.70×10^-4^ | 0.404 | 0 |
| CL4 | 1.32×10^-5^ | 1.42×10^-5^ | 0.929 | 0 |

^1^Variance ratio test testing deviation from 1.

**Additional File 1. Figure 4**. Distributions of estimates of homozygosity (A and B) and relatedness within clusters (C and D) when using cluster specific estimates of frequencies (A and C) versus global frequencies (B and D).

**Possibly Damaging Rare Variants (PDV) in PAGES Sample.**

Using the results in Mahjani et al. (in review) and their classification scheme A, we tabulate the variation observed in the PAGES sample matching our criterion for possibly damaging variation, which we call PDV variants (Table 4). We note, however, that we removed subjects carrying large chromosomal abnormalities, the majority of whom were trisomy of chromosome 21. This trisomy invariably generates the distinct phenotype of Down’s Syndrome (DS). DS is comorbid with ASD more than expected by chance[6]. Still, given the substantial number of DS subjects in the collection (N=12), we suspected the inclusion of this syndrome would have an outsize impact on the study. We likewise believed that we should treat other carriers of very large CNVs equivalently to our treatment of chromosome 21 trisomies. We likewise removed subjects whose DNA suggested the presence of multiple CNVs because this multiplicity is an indication of false positives resulting from poor quality DNA.

| **Additional File 1: Table 4**. Treatment of possibly damaging variants from the PAGES subjects diagnosed with AD. “ASC 102” refers a variant falling into one of the 102 inferred ASD genes reported in Satterstrom et al. | | | |
| --- | --- | --- | --- |
|  | CNV | PTV^3^ (ASC 102) | MIS^4^ (ASC 102) |
| Initial count | 108 | 23 | 32 |
| Unique subjects | 96^1^ | 23 | 28 |
| After Selection^2^ | 88 | 20 | 23 |

^1^Mahjani et al. (in review) report one more carrier (16p11.2 deletion). This observation was removed during our QC. ^2^The data we analyze here are a subset of the data analyzed in Mahjani and were selected on the basis of European ancestry, same genotyping platform per case status, and quality control metrics on samples and genotypes. Our analyzed samples differ slightly from those in Mahjani because we emphasize different features of QC (i.e., European ancestry versus CNV calls for Mahjani). ^3^Protein truncation variant. ^4^Missense variant.

**Effectiveness of Matching and Impact of Correction for Principal Components of Ancestry.**

If genetic matching was heterogeneous over clusters, we would expect that GP would differ among them. We find no evidence for heterogeneity among clusters (Table 5). Curiously, however, the model that removes the effects of the 10 largest EVAs does induce a small but significant difference among clusters (Table 5). Accompanying this effect is a small but significant reduction in the ability to distinguish ASD versus unaffected status of pairs of individuals by GP (Table 6). We believe it likely that later EVAs are removing heritable variation related to ASD status. This conjecture, however, cannot be proven by this experiment.

**Additional File 1: Table 5.** Analysis of variance of GP as a function of ancestry cluster (CLS), diagnosis using different numbers of ancestry eigenvectors (EVA) in the genomic prediction model.

| EVA | p-value for CLS |
| --- | --- |
| 0^1^ | 0.497 |
| 0^2^ | 0.496 |
| 3^2^ | 0.536 |
| 10^2^ | 0.019 |

^1^Based on the pair-out algorithm, ^2^Based on the one-out algorithm

**Additional File 1: Table 6.** Logistic regression of ASD status on GP and carrier status GP (cases only) using different numbers of ancestry eigenvectors (EVA) in the genomic prediction model.

|  | ASD status | | | | PDV carrier status | | |
| --- | --- | --- | --- | --- | --- | --- | --- |
| EVA | OR | 95%CI | P | Pseudo-R^2^ (%) | OR | 95%CI | P |
| 0^1^ | 1.67 | 1.58-1.77 | 6.73×10^-32^ | 7.80 | 0.81 | 0.71-0.92 | 8.36×10^-4^ |
| 0^2^ | 1.66 | 1.57-1.76 | 2.65×10^-31^ | 7.64 | 0.81 | 0.71-0.92 | 8.56×10^-4^ |
| 3^2^ | 1.67 | 1.58-1.76 | 1.65×10^-31^ | 7.69 | 0.81 | 0.71-0.92 | 8.15×10^-4^ |
| 10^2^ | 1.60 | 1.51-1.69 | 1.80×10^-30^ | 6.60 | 0.81 | 0.72-0.91 | 3.87×10^-4^ |

^1^Based on the pair-out algorithm, ^2^Based on the one-out algorithm

**Other Approaches to Normalizing Allele Frequencies.**

Here we explore the effect of estimating allele frequency in two other ways than the one used in the main manuscript, the CLS (within-cluster standardization). These other approaches are more typical of the methods described in the literature (Table 7): 1) estimating allele frequencies from the combined set of unmatched unaffected subjects (POP), and 2) estimating the allele frequencies from the entire, combined sample of subjects (GCTA). Both approaches lead to a small bias in estimates of GP (Table 7). We also explore a variant of the GP estimation plan, rather than leaving out the matched pair and jointly estimating GP, we leave out only one subject and estimate GP, finding that this slight difference in sampling generates similar estimates of GP (Table 7).

**Additional File 1: Table 7.** Anova results of GP as a function of cluster (CLS) and logistic regression results of GP as a function of DX when using different GRMs.^1^

| GRM | P(CLS)^4^ | P(GP)^5^ |
| --- | --- | --- |
| CLS^2^ | 0.497 | 6.73×10^-32^ |
| CLS^3^ | 0.496 | 2.65×10^-31^ |
| POP^3^ | 0.006 | 3.89×10^-31^ |
| GCTA^3^ | 0.006 | 4.96×10^-31^ |

^1^ Genomic prediction performed without adjusting for ancestry, ^2^ Based on the pair-out algorithm, ^3^Based on the one-out algorithm, ^4^Anova results, ^5^Logistic regression results.

We next asked whether the effect of choosing different approaches to estimating population-level allele frequencies can be removed by using ancestry covariates (EVA or PCA) in the estimation model (MME). For POP, we chose to use 3 EVA. For GCTA, we chose the standard 10 PCA, the number often used in analysis of genomic data when using the GCTA software (Table 8). Using POP-GRM with 3 EVA results in a very strong cluster (CLS) effect on GP. Adding 10 PCA to the MME when using GCTA-GRM does control the effect of CLS on GP (p = 0.713). It does, however, come at a cost of less accurate prediction for ASD diagnosis status from GP (OR = 1.54; 95%CI = 1.46-1.62; P = 4.52×10^-24^; pseudo-R^2^ = 5.68%).

**Additional File 1: Table 8.** Anova results of GP as a function of cluster (CLS) and logistic regression results of GP as a function of DX when using different GRMs but accounting for difference in ancestry by using ancestry vectors as covariates in the genomic prediction model.

| GRM | Ancestry Covariates | P(CLS)^3^ | P(DX)^4^ |
| --- | --- | --- | --- |
| CLS^1^ | None | 0.497 | 6.73×10^-32^ |
| CLS^2^ | None | 0.496 | 2.65×10^-31^ |
| POP | 3 EVA | 1.63×10^-20^ | 4.17×10^-31^ |
| GCTA | 10 PCA | 0.713 | 4.52×10^-24^ |

^1^Based on the pair-out algorithm, ^2^Based on the one-out algorithm, ^3^Anova results, ^4^Logistic regression results.

**Training-Testing.**

We used a pair-out (or single-out) approach to the training-testing algorithm, a computer intensive approach for calculation of the G-BLUP solutions for GP. A reasonable question is whether a more traditional and computationally less intensive training-testing – splitting data in larger subsets – would yield similar results. For this experiment, we randomly split the data in folds or splits and used a portion for training and the remainder for testing (Table 9-10). Because these splits are random, we repeated this process 25 times. Notably, as the number of splits increases, accuracy approaches the two- or one-out approach, so careful selection of fold size could expedite calculations with only minor loss in accuracy.

**Additional File 1: Table 9.** Average results from the ANOVA of the prediction of GP from CLS when using different number of splits for of the data for training and testing (25 repetitions).

| Splits | P(CLS) |
| --- | --- |
| 2 | 0.298 |
| 4 | 0.365 |
| 10 | 0.487 |
| 20 | 0.449 |
| One-out | 0.496 |
| Pair-out | 0.497 |

**Additional File 1: Table 10.** Average results for the logistic regression of ASD status on GP and PDV status in cases on GP when using different splits of the data for training and testing 25 repetitions).

|  | ASD status | | | | PDV carrier status | | |
| --- | --- | --- | --- | --- | --- | --- | --- |
| Splits | OR | 95%CI | P | Pseudo-R^2^ (%) | OR | 95%CI | P |
| 2 | 1.50 | 1.42-1.58 | 4.24×10^-18^ | 5.02 | 0.86 | 0.76-0.97 | 0.0379 |
| 4 | 1.58 | 1.50-1.67 | 1.16×10^-23^ | 6.35 | 0.83 | 0.74-0.94 | 0.0076 |
| 10 | 1.64 | 1.55-1.73 | 5.70×10^-28^ | 7.23 | 0.82 | 0.72-0.93 | 0.0021 |
| 20 | 1.66 | 1.57-1.75 | 4.03×10^-30^ | 7.60 | 0.82 | 0.72-0.92 | 0.0016 |
| One-out | 1.66 | 1.57-1.76 | 2.65×10^-31^ | 7.64 | 0.81 | 0.71-0.92 | 8.56×10^-4^ |
| Pair-out | 1.67 | 1.58-1.77 | 6.73×10^-32^ | 7.80 | 0.81 | 0.71-0.92 | 8.36×10^-4^ |

**Case-Control Balance**

**Additional File 1: Figure 5**. Case-control balance. Results of 100 simulations of different degrees of imbalance and showing the distribution of p-values for a cluster effect on burden. (A) A balanced, *but not matched* design of 300 cases and 300 controls for each of four ancestry clusters; (B) An unbalanced design in which 300 ASD subjects per cluster were contrasted with a varying, but unbalanced number of unaffected subjects (330, 315, 285, or 270 unaffected subjects, randomly assigned per cluster and per simulation); (C) The unbalanced design of (B) but using three eigenvectors of ancestry to account for differences among ASD and unaffected subjects.

**Analysis by Cohort.**

To determine how GP’s ability to distinguish ASD from unaffected individuals varied on cohort, we analyzed cohorts separately (Table 11). GP is a significant predictor for both cohorts, although it is less accurate for PAGES than for SSC. This is consistent with the less complete characterization of PAGES for molecular assays (WES and CNV calling) and *de novo* status.

**Additional File 1: Table 11**. Results from the case-control comparison when data were analyzed by the origin of the cases in each matched pair.

| Origin | Pairs | OR | 95%CI | P | Pseudo-R^2^ (%) |
| --- | --- | --- | --- | --- | --- |
| PAGES | 1,015 | 1.58 | 1.44-1.73 | 6.16×10^-10^ | 6.33 |
| SSC | 1,996 | 2.27 | 2.11-2.45 | 1.61×10^-42^ | 17.01 |

**NULL Distribution.**

By estimating GP for each pair of subjects, it is reasonable to expect some overfitting of the predictions and inflation of the test-statistic when the distributions of GP for individuals diagnosed with ASD versus unaffected individuals are compared. We examined the distribution of the test statistic using the following experiment. From each cluster we randomly chose the same number of samples as there were affected samples in the cluster. These were then pair-matched to the remaining samples in the cluster. We then randomly assigned “affected” and “unaffected” status within each pair. Based on these assignments, we subsequently calculated GP and used logistic regression to test how well the GP predicted “affection” status. This matched our original ASD and unaffected-pair GP analysis exactly. We repeated this process 256 times. The distribution of p-values and test-statistic showed a shift in p-values toward zero, which is due to over-dispersion of the test statistic (mean is not significantly different from 0, p =0 .61). This shift is easy to address by either Genomic Control or GC (λ = 2.406)[7] or by using the empirical variance of the test-statistic (var = 2.255). Using either adjustment, we can obtain the proper size of the test. Here we use GC to adjust the p-values for case-control comparisons for GP and WGRS (Fig. 6) for all relevant p-values reported in the main manuscript and Additional File 1.

We also randomly assigned PDV carrier status to the “affected” samples in each of simulations. Again, we used the same distribution of the carrier status across clusters as in the original data. Based on these assignments we ran a logistic regression with PDV status as the outcome. Of the 256 repetitions there were only four with a p < 0.05 (expectation=12), showing no need for adjustments when comparing the PDV carrier status.

**Additional File 1: Figure 6**. Distribution of p-values and test statistics before (A, B) and after (C, D) correction using Genomic Control (GC).

**Quality Control for SNPs Selected for PRS Calculations and Motivation for Threshold.**

SNPs from autism spectrum disorder[8], schizophrenia[9], and educational attainment[10] GWAS were selected as follows: (1) Select GWAS SNPs that were part of our imputed and QC-ed set of 5,145,175 SNPs. (2) Remove any SNP whose minor allele frequency MAF – as estimated from the control samples used for frequency estimation in our data – deviates by more than 0.075 from the GWAS-reported allele frequency. (3) Remove palindromic SNPs with MAF > 0.40 in our data. (4) Remove SNPs in the GWAS data with MAF < 0.005. (5) Take only the most significant SNP in the MHC region. Remove all other SNP in the region chr6:25,000,000-34,000,000 Mb. (5a) For EA, there are an additional 12 regions with a concentration of SNP with small p-values where the smallest p-value in the region < 1×10^-24^. Even though the markers in these regions might not be in high LD with each other (r^2^ < 0.50, imposed in step (7)), they are still likely to be the result from a single causal variant in the region. We therefore opted to use only the most significant SNP from each of the following regions for calculating PRS for EA: chr1:4,370,000-4,455,0000; chr1:72,400,000-73,000,000; chr1:204,350,000-204,650,000; chr2:100,100,000-101,400,000; chr3:48,500,000-50,500,000; chr5:59,700,000-60,900,000; chr5:87,300,000-88,300,000; chr6:98,000,000-99,000,000; chr9:23,340,000-23,450,000; chr12:123,400,000-124,000,000; chr13:58,200,000-58,800,000; and chr18:35,000,000-35,500,000; (6) Clump SNPs using --clump in PLINK based on the p-values from the GWAS and the LD structure calculated from the control samples used for frequency calculations in our data (setting: clump-r^2^ = 0.50 and clump-kb = 50). (7) Use SNPs with clump p-value < 0.01. With SNPs selected by this procedure, PRS was calculated using --score in PLINK with option center. Centering of genotype counts was based on the frequencies in the controls that were not part of the matches in our data by using PLINK option --read-freq.

The motivation for our choice of threshold 0.01 follows: (1) We wanted the PRS to carry information, yet to be largely independent of the GP estimate, so it should be relatively sparse in terms of SNPs. The 0.01 threshold met this criterion. (2) Also, although studies using a PRS often explore multiple p-value thresholds, we wanted to limit it to one, which limits the number of hypothesis tests to one and thereby obviates concerns about multiple testing and corrections. Looking over the optimal parameter solutions presented in *Making the Most of Clumping and Thresholding for Polygenic Scores* [11], which explores optimization of this and other parameters for nine GWAS, the median estimate was 0.01. Hence, we chose this threshold, which corresponds to the optimal threshold for type-2 diabetes.

After calculation of ASD PRS for parents and siblings in the SSC data, we determined pTDT for the children (probands and siblings)[12]. Correlation between ASD-PRS and pTDT_ASD_ was 0.721 (p = 0). Results show that, on average, pTDT_ASD_ for ASD-PDV are similar to those for the SIBLING group, while pTDT_ASD_ for ASD-NO-PDV indicate an increased ASD risk burden over the SIBLING (Table 12).

**Additional File 1: Table 12**. pTDT_ASD_ for different subsets of children in the SSC dataset

| Subset | N | Mean | SD | p-value |
| --- | --- | --- | --- | --- |
| ASD-NO-PDV | 1814 | 0.153 | 0.985 | 4.66×10^-11^ |
| ASD-PDV | 174 | -0.011 | 1.109 | 0.900 |
| SIBLING | 1637 | -0.023 | 0.994 | 0.355 |

We also investigated the PRS for educational attainment (EA). Using the GWAS results from Lee et. al. 2018 [10], we performed pruning and thresholding as described above, resulting in a PRS based on 39,742 SNP. Odds ratio for ASD versus unaffected status using the ED-PRS is 1.09 (p=0.001; 95%CI:1.03-1.14; pseudo-R^2^: 0.24%). Differentiating ASD individuals with and without possibly damaging mutations using EA resulted in a non-significant OR of 0.94 (p = 0.279; 95%CI: 0.84-1.05; pseudo-R^2^: 0.08%).

**Alternative Weighting.**

There are many ways to combine scores. For instance, we combined GP, ASD-PRS, and SCZ-PRS using their relative pseudo-R^2^ as weights, specifically 78.2%, 12.3%, and 9.5% for GP, ASD-PRS, and SCZ-PRS, respectively. As described in Table 3 of the main manuscript, this produced a WGRS with an odds ratio of 1.73 (95% CI: 1.64-1.83).

Here we present results from two alternative weighting schemes: 1) equal weights; and 2) an optimal weighting scheme based on training/testing of the data. (Following “A K-fold Averaging Cross-validation Procedure”[13]). For the training/testing we divided the data into 5 folds: for each fold, we then trained using the remaining folds to determine betas from the logistic regression model in which ASD status is predicted from GP, PRS-ASD, and SCZ-PRS. For the fold of interest, we calculated a WPRS^*^ and determined how well this predicts ASD status in the fold of interest as measured by the log_10_(P). After all folds were processed, we then took a weighted average of the betas with weights based on log_10_(P) to determine the final betas used for combining the three scores (Table 13). This scheme puts slightly less emphasis on GP and increases the emphasis on the two PRS. After applying these weights to the complete data and analyzing case-control status, we obtain OR = 1.75; 95%CI = 1.66-1.85; P = 1.43×10^-36^, a slight although non-significant improvement over the scheme that we chose *a priori* for our analysis. Similarly, we see a slight improvement when analyzing PDV carrier status (OR = 0.74; 95%CI = 0.65-0.84; P = 3.02×10^-6^) over our *a priori* weighting from Table 3 (OR = 0.77; 95%CI = 0.68-0.87; P = 4.38×10^-5^). Applying equal weights yields OR = 1.63; 95%CI = 1.54-1.72; P = 3.83×10^-29^ for the case-control difference and OR = 0.72; 95%CI = 0.63-0.82; P = 2.82×10^-7^ for the PDV carrier status.

**Additional File 1:** Table 13. Results from the five folds to determine the weights for combining GP, ASD-PRS, and SCZ-PRS.

|  |  | Beta |  |  |  |
| --- | --- | --- | --- | --- | --- |
| Fold | GP | ASD-PRS | SCZ-PRS | -log10(P) | weight |
| 1 | 0.505 | 0.164 | 0.141 | 14.729 | 0.166 |
| 2 | 0.506 | 0.182 | 0.159 | 11.638 | 0.131 |
| 3 | 0.467 | 0.162 | 0.116 | 24.041 | 0.271 |
| 4 | 0.491 | 0.160 | 0.133 | 19.901 | 0.224 |
| 5 | 0.497 | 0.162 | 0.108 | 18.505 | 0.208 |
| Weighted average | 0.490  (62.6%) | 0.162  (21.0%) | 0.108  (16.4%) |  |  |

**PDV Carriers Intermediate Between Controls and Non-Carriers.**

Here we evaluate whether the observation that GP for ASD-PDV is intermediate between ASD-NO-PDV and unaffected individuals is due to our methodology, as opposed to something inherent in the samples. To do so, we performed the following experiment. Replace the matched pairs in which the cases are PDV carriers and replace them with matched control pairs, in which one of each pair functions as a pseudo-PDV carrier. We then calculated GP on the complete set of matched pairs. This process was repeated 256 times and we recorded the mean for each of the three categories over repetitions. Means and standard error were -0.231 (0.0005), -0.179 (0.0034), and 0.246 (0.0004) for the unaffected, pseudo-PDV and non-carrier individuals, respectively. The pseudo-PDV carrier samples have mean GP near the unaffected, rather than intermediate between unaffected and non-carrier individuals, as is seen in the true experiment.

**Removing CNVs that have less certain impact on risk.**

As we note in our Limitations section, we cannot be certain all of the variants labeled PDVs actually carry risk for ASD. In final review an editor asked for a set of pdCNVs to be re-evaluated for this reason. We thus redid our calculations removing these pdCNVs, which removes 28 ASD PDV carriers from our analyses (Table 14).

**Additional File 1:** Table 14. WGRS summary statistics for different subsets of the samples after removing 28 subjects who carried these CNVs with greater uncertainty about PDV status.

|  |  | WGRS | | P | |
| --- | --- | --- | --- | --- | --- |
| Subset | N | Mean | SE | vs Unaffected | vs ASD-NO-PDV |
| Unaffected | 3011 | -0.265 | 0.017 |  |  |
| ASD-CNV | 166 | -0.020 | 0.069 | 0.053 | 7.79×10^-05^ |
| ASD-CNV-ALT^1^ | 138 | -0.010 | 0.076 | 0.065 | 4.64×10^-04^ |
| ASD-PTV | 78 | 0.034 | 0.112 | 0.099 | 0.024 |
| ASD-MIS | 61 | 0.237 | 0.115 | 0.013 | 0.731 |
| ASD-PDV | 305 | 0.045 | 0.053 | 0.001 | 4.38×10^-05^ |
| ASD-PDV-ALT^1^ | 277 | 0.057^2^ | 0.056 | 0.001 | 1.99×10^-04^ |
| ASD-NO-PDV | 2682 | 0.279 | 0.018 | 6.08×10^-36^ |  |

^1^ Reflects that an additional 28 CNV carrier samples that were re-evaluated per the editor’s request were removed.

^2^ This value would replace the $\bar{x}_{ASD-PDV}$ in Figure 4C when using the narrower definition of CNV.

**Literature Cited.**

1. de los Campos G, Gianola D, Allison DB. Predicting genetic predisposition in humans: the promise of whole-genome markers. Nature reviews Genetics. 2010;11(12):880-6.

2. Henderson CR. Best linear unbiased estimation and prediction under a selection model. Biometrics. 1975;31(2):423-47.

3. Chaste P, Klei L, Sanders SJ, Hus V, Murtha MT, Lowe JK, et al. A genome-wide association study of autism using the Simons Simplex Collection: Does reducing phenotypic heterogeneity in autism increase genetic homogeneity? Biol Psychiatry. 2015;77(9):775-84.

4. Lee AB, Luca D, Klei L, Devlin B, Roeder K. Discovering genetic ancestry using spectral graph theory. Genet Epidemiol. 2010;34(1):51-9.

5. Lee AB, Luca D, Roeder K. A Spectral Graph Approach to Discovering Genetic Ancestry. Ann Appl Stat. 2010;4(1):179-202.

6. DiGuiseppi C, Hepburn S, Davis JM, Fidler DJ, Hartway S, Lee NR, et al. Screening for autism spectrum disorders in children with Down syndrome: population prevalence and screening test characteristics. J Dev Behav Pediatr. 2010;31(3):181-91.

7. Devlin B, Roeder K. Genomic control for association studies. Biometrics. 1999;55(4):997-1004.

8. Grove J, Ripke S, Als TD, Mattheisen M, Walters RK, Won H, et al. Identification of common genetic risk variants for autism spectrum disorder. Nature genetics. 2019;51(3):431-44.

9. Schizophrenia Working Group of the Psychiatric Genomics C. Biological insights from 108 schizophrenia-associated genetic loci. Nature. 2014;511(7510):421-7.

10. Lee JJ, Wedow R, Okbay A, Kong E, Maghzian O, Zacher M, et al. Gene discovery and polygenic prediction from a genome-wide association study of educational attainment in 1.1 million individuals. Nature genetics. 2018;50(8):1112-21.

11. Privé F, Vilhjálmsson BJ, Aschard H, Blum MGB. Making the Most of Clumping and Thresholding for Polygenic Scores. American journal of human genetics. 2019;105(6):1213-21.

12. Weiner DJ, Wigdor EM, Ripke S, Walters RK, Kosmicki JA, Grove J, et al. Polygenic transmission disequilibrium confirms that common and rare variation act additively to create risk for autism spectrum disorders. Nature genetics. 2017;49(7):978-85.

13. Jung Y, Hu J. A K-fold Averaging Cross-validation Procedure. J Nonparametr Stat. 2015;27(2):167-79.
